# The Association Between French Veterinary Practice Characteristics and Their Revenues and Veterinarian's Time Use

**DOI:** 10.3389/fvets.2021.675028

**Published:** 2021-06-11

**Authors:** Ikram Abdouttalib, Youba Ndiaye, Ahmed Ferchiou, Didier Raboisson, Guillaume Lhermie

**Affiliations:** ^1^CIRAD, UMR ASTRE, Montpellier, France; ^2^ASTRE, CIRAD, INRAE, Univ Montpellier, Montpellier, France; ^3^Université de Toulouse, ENVT, Toulouse, France; ^4^Department of Production Animal Health, University of Calgary, Calgary, AB, Canada

**Keywords:** veterinary practice, profitability, economics, companion animals, food-producing animals

## Abstract

The provision of healthcare by veterinarians consists of a blend of activities ensuring welfare for animals. It also contributes in the control of infectious diseases and food safety. In general practices, most of the activities generate incomes for veterinarians, notably acts (consultations, surgery, etc.) and sales (drugs, pet food, etc.). Increased size of veterinary practices and the arrival of corporate companies modify the veterinary landscape in many countries. In a context of rapid growth of the companion animal health market, the question of the profitability of veterinary activities is relevant. Indeed, beyond a certain threshold, veterinarians may be tempted to leave behind food-producing animals' acts and focus on companion animals' acts, which are generally recognized to be more profitable and more attractive for new generations of veterinarians. A survey was conducted in French veterinary mixed practices, and a regression analysis was used to quantify the relationships between the turnover and the characteristics of veterinary practices, the time to perform veterinary acts, and the characteristics of veterinarians. We found that the characteristics of veterinary practices are positively associated with the turnover and the price of acts, and that there was an association between the status of veterinarians (associate, collaborator, or employee) and the time required to perform companion animals' and food-producing animals' acts. The present study is the first study showing the association between the characteristics of veterinary practices and the turnover, by investigating the price of veterinary acts and the time required.

## Introduction

The provision of healthcare by veterinarians consists of a blend of activities ensuring welfare for animals, control of infectious diseases, and food safety. In France, as in many countries, animals' owners primarily pay services provided by veterinarians. In general practices, a diversity of activities generate incomes for veterinarians, notably acts and sales. Healthcare provision can be divided in two main sectors: companion animal (CA) and food-producing animal (FPA). It is noteworthy that at the practice and veterinarian level, many practices or individuals endorse a status of so-called mixed, meaning that they exert both CA and FPA medicine and surgery.

In the CA market, the veterinarians sell drugs, consultations, pet foods, and other types of goods. In the FPA market, veterinarians also exert an activity of population medicine and sanitary surveillance under a public mandate.

The French veterinary sector generated a turnover of 3.5 billion euros excluding taxes in 2016. The turnover of this sector increased by 3.6% in value over the period 2000–2016 (1). This increase was driven by the CA acts (+4.8% on average) over the period, and the share of veterinary expenditure devoted to CA passes from 0.17 to 0.24% of household budgets (1).

In a context of rapid growth in the CA health market and decline in the FPA health market, the question of the profitability of veterinary activities is relevant. Indeed, beyond a certain threshold, veterinarians may be tempted to leave behind FPA activities and focus on CA activities, which are generally seen to be more profitable (2) and more attractive for new generations of veterinarians. This may lead to shortages of veterinarians in the FPA sector, threatening sanitary surveillance and accessibility of veterinarians for some farmers. Even if, from a societal perspective, this situation is not desirable, the veterinary market is primarily a private market, and therefore, veterinary practice is a firm whose purpose is to produce or provide goods or services to a set of clients, the associates of the veterinary practice, given their rational behavior, seeking to maximize their profit.

In a recent publication, we calculated the average profitability through the net margin rate, expressed as the ratio profit over revenue, by comparing inputs and outputs (2). This first approach enables to compare the performance of veterinary practices. Yet, optimal resource allocation requires to evaluate the marginal profitability of each activity given a context, and not the average one, to inform short- and long-term projection of the practice business model. The calculation of the marginal profitability is limited by time required for each activity, and there is a high inconsistency observed in the field or when asking field actors.

Performance management plays a central role health service structures as in the development of hospital management and healthcare performance assessment (3). An efficient and rational healthcare performance measurement system can improve medical service quality, reduce costs, optimize service processes (4), and achieve optimal resource allocation (5).

Time required for each activity is also recognized as a key factor limiting prediction in agriculture (6) and more broadly in biology (7). The situation is all the more complex for veterinarians that they performed a high range of activities in a high diversity of contexts (8). Factors influencing the time required for veterinary activities are the nature of the act, the species, the context (on farm/at home intervention), and the way it is invoiced (per act, all-inclusive, etc.).

The aim of this present study is to analyze the association between the characteristics of veterinary practices and the turnover, by investigating the price of veterinary acts and the time required.

## Materials and Methods

### Data

**Figure 1:**
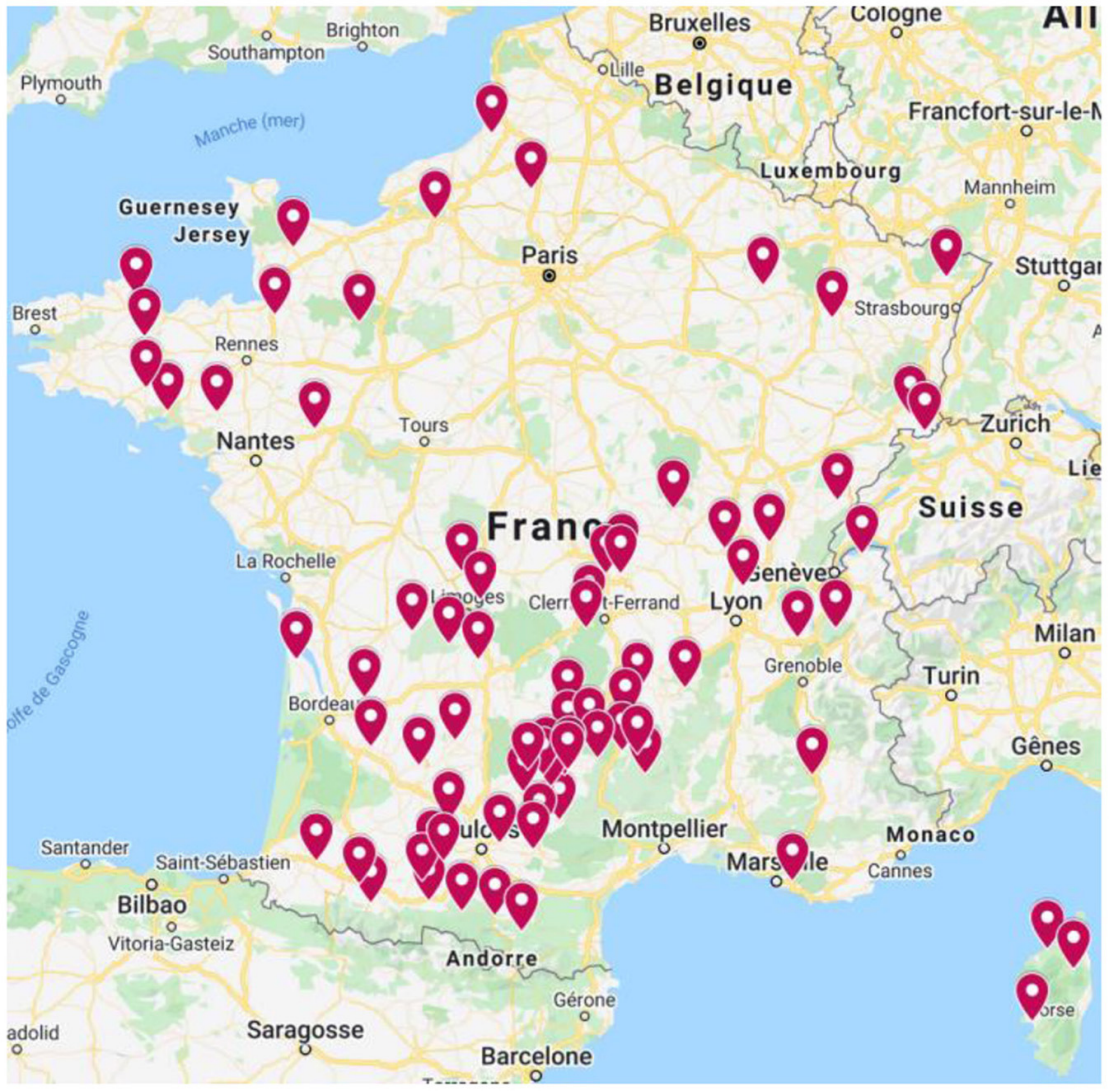
Breakdown of responding veterinarians in Metropolitan France (9).

A two-part survey questionnaire was developed and administered in person in January 2020. Veterinarians (associates or employees) working in mixed practices (CA and FPA acts) in France were asked a set of 36 questions arranged in four blocks: structure of the practice, prices of veterinary acts performed in this practices, individual information describing the respondent's profile, and time spent by each respondent to perform veterinary acts. The number of participants in the survey was 96, spatially distributed as shown in the Figure 1.

Answers from the survey were arranged in a database regrouping information provided by 96 respondents and 14 variables.

### Variables Used in Statistical Analysis

The data includes 10 variables related to the characteristics of veterinary practices (Table 1): turnover, number of full-time equivalent (FTE) associates, number of FTE employees, percentage of CA acts, percentage of FPA acts, percentage of equine acts, surface, practice working hours, year of opening the practice, and the invoiced price for veterinary acts.

**Table 1:** Definition of variables characteristic of veterinary practices and characteristic of veterinarians used in statistical analysis.

**Variable**	**Unit**	**Definition**	**Characteristic of the veterinary practice**	**Characteristic of the veterinarian surveyed**	**PCA**	**Regression**
Turnover	€	Sum of the annual receipts generated by the sale of drugs, material, and veterinary acts	+	–	+	+
FTE associates	FTE	Number of veterinarians linked to one or more associates by a community of interest and in particular who has a part of capital; an associate is equivalent to one FTE, i.e., he works full-time (10)	+	–	+	+
FTE employee	FTE	Number of veterinarians employed by the associates who undertakes to perform veterinary acts, in return for a salary. An employee works an average of 35 h per week, which is equivalent to 0.8 FTE (10)	+	–	+	+
Ratio of CA–FPA acts	None	The percentage of veterinary activities dedicated to CA acts divided on the percentage of veterinary activities dedicated to FPA acts	+	–	–	+
Percentage of the veterinary activity	%	The percentage of CA acts, FPA acts, and equine acts	+	–	+	+
Surface	m^2^	The surface of the veterinary practice composed by consultation rooms and surgery rooms	+	–	+	+
Working hours	Hours/week	Sum of weekly opening hours of the veterinary practices	+	–	+	+
Opening year	None	An indicator of the age of the veterinary practices	+	–	+	–
Weighted price	€	An indicator that represents the variable that summarizes all the information on the prices of the veterinary acts declared by each practice	+	–	–	+
Weighted time	Minutes	An indicator that represents the variable that summarizes all the information on the times required to perform different veterinary acts declared by each veterinarian surveyed	–	+	–	+
Status	None	A qualitative variable composed of three modalities: associate, collaborator, and employee	–	+	–	+
Class year experience	Year	Variable representing the experience of each veterinarian surveyed. The year of experience variable was calculated from the year of graduation declared by the veterinarians (year of experience = 2020 – year of graduation). This variable was converted into four classes : (i) < 5 years of experience, (ii) from 5 years included to 10 years included, (iii) from 10 to 15 years included, and (iv) more than 15 years	–	+	–	+
Gender	None	The gender of the veterinarian composed of two modalities, male or female	–	+	–	+
Median of the standard of living	€/consumption units	The disposable income for household in the practice area divided by the number of consumption units (1)	Other	Other	–	+

*+, the variable was considered as/in; –, the variable was not considered as/in; other, the variable was considered as a characteristic of the communes, where the veterinary practices of the sample are located*.

In addition to the variables characterizing veterinary practices, the database also consists of variables characterizing veterinarians surveyed, who may be alternatively associates, employees, or collaborators. These variables capture their status, gender, age, year of completion of studies, and the time of veterinary acts.

The weighted price and weighted time of veterinary acts were calculated, by weighting the prices and times reported in the surveys, since they are highly variable, and in order to obtain a single variable that summarizes all of this information. Among the price for all CA acts, six acts were selected since they are the most frequent. In order to calculate the weighted price for six acts, a coefficient was first calculated and assigned to each of the price; this coefficient represents the ratio between the average of the price of an act on the total price, and then, the weighted price was calculated and represents the sum of the price of each veterinary acts declared by each veterinary practices multiplied by the coefficient corresponding to each act.

Since there are some veterinary acts that take more time than others do, the weighted times to perform CA acts and FPA acts were calculated by applying the same method used to calculate the weighted price. Among the time for all CA acts, the six most frequent acts were selected. In order to calculate their weighted time, a coefficient was first calculated and assigned to each of the time. This coefficient represents the ratio between the averages of the time of an act on the total time. The weighted time was afterwards calculated; it represents the sum of the time of each veterinary acts declared by each veterinary practices multiplied by the coefficient corresponding to each act.

### Descriptive Statistics

Data were first recorded in Microsoft Excel datasheet and further analyzed using R software, version 3.3.3 (11), to quantify the associations between French veterinary practice characteristics and their revenues and veterinarians' time. The descriptive statistics of the quantitative and qualitative variables are presented in Tables 2, 3.

**Table 2:** Descriptive statistics of quantitative variables used in regression models and PCA.

	**Mean**	**Std. dev**	**Min**	**Max**
Log (turnover)	13.91	0.49	12.39	15.20
Percentage of CA acts (%)	44.67	16.68	10.02	80.10
Percentage of FPA acts (%)	46.77	18.19	5.06	95.15
Ratio of CA–FPA acts	0.74	1.78	0.02	1.00
Surface (m^2^)	244.25	129.95	55.90	1,000
Working hours (hours/week)	52.46	4.79	35.67	63.14
FTE associate (FTE)	2.83	1.68	1.12	7.04
FTE employee (FTE)	2.00	1.50	0.02	7.21
Log (CA weighted price)	3.51	0.18	3.09	4.09
Log (FPA weighted price)	4.55	0.21	3.90	5.06
Median level life (€)	20,075	1,696	16,040	249,80
CA weighted time (minutes)	32.22	20.49	22.56	62.46
FPA weighted time (minutes)	34.30	11.10	29.34	155.06

*PCA, principal component analysis; CA, companion animal; FPA, food-producing animal; FTE, full-time equivalent*.

**Table 3:** Summary statistics of qualitative variables (characteristic of the veterinarians surveyed).

**Gender**		**Status**			**Class of year experience**			
**Female**	**Male**	**Associate**	**Employee**	**Collaborator**	** <5 years**	**≥5 and ≤10 years**	**>10 and ≤15 years**	**>15 years**
32	64	62	30	4	28	17	11	40

A principal component analysis (PCA) was performed followed by a hierarchical cluster analysis (HCA). The multivariate analysis was performed with the “FactomineR” package (12). The HCA used Ward's method and was consolidated with the *K*-means method. HCA with Ward's method is a clustering method that identifies groups of points in the Euclidian space represented in the PCA (12).

The variables introduced in this descriptive analysis have been grouped in three blocks: economic performance (turnover, FTE associate, and FTE employee), nature of the veterinary practice (percentage of CA acts, percentage of FPA acts, and percentage of equine acts), and the structure of the veterinary practice (surface, working hours, and opening year).

### Regression Analysis

First, a regression model was performed to show the association between the turnover and the variables characteristic of veterinary practices in the following Equation (1). The dependent variable *Yi* represents log (turnover) declared of 96 veterinary practices, and independent variables *Xi* represents the characteristics of the veterinary practices (FTE associate, FTE employee, surface, working hours, and the ratio of CA–FPA acts):

(1) $$\begin{array}{l} \text{Log }\left(\text{Turnover}\right)=\beta_{1}\text{ FTE}\\ \text{ \_associate}+\beta_{2}\text{ FTE}\\ \text{ \_employee}+\beta_{3}\text{ Surface}+\beta_{4}\text{ Working}\\ \text{ \_hours}+\;\beta_{5}\text{ Ratio\_CA}\\ \text{ \_FPA\_acts}+\;\epsilon \end{array}$$

Then, two sub-models were applied; in the first one, the percentage of CA acts was integrated instead of the ratio of CA–FPA acts, and the percentage of FPA acts instead of the ratio of CA–FPA acts in the second.

Second, two models were applied to analyze the association between the weighted price and the characteristic of veterinary practices. In the first model (Equation 2), the dependent variable (*Yi*) represents log (weighted price of CA acts), and in the second model (Equation 3), the dependent variable (*Yi*) is log (weighted price of FPA acts). The explanatory variables used in the models are (i) the variable characteristic of the veterinary practices (FTE associate, FTE employee, surface, working hours, and the ratio of CA–FPA acts) and (ii) the median of the standard of living of each of the communes (where the veterinary practices of the sample are located), in order to see the effects of wealth on the willingness to pay for veterinary acts.

(2) $$\begin{array}{l} \text{Log }\left(\text{weighted price of CA acts }\right)=\beta_{1}\text{ FTE\_associate}\\ \;+\beta_{2}\text{ FTE\_employee}\\ \;+\beta_{3}\text{ Surface}+\beta_{4}\text{ Working}\\ \text{ \_hours}+\;\beta_{5}\text{ Ratio\_CA\_FPA}\\ \text{ \_acts}+\beta_{6}\text{ Median\_standard}\\ \text{ \_living}+\;\epsilon \end{array}$$

(3) $$\begin{array}{l} \text{Log }\left(\text{weighted price of FPA acts }\right)=\;\beta_{1\;}\text{FTE associate}\\ \;+\beta_{2\;}\text{FTE employee}\\ \;+\beta_{3\;}\text{Surface}\\ \;+\beta_{4\;}\text{Working hours}\\ \;+\;\beta_{5}\text{ Ratio\_CA\_FPA\_acts}\\ \;+\beta_{6}\text{ Median\_standard\_living}\\ \;+\;\epsilon \end{array}$$

Third, two linear regression models were used to analyze the association between the weighted time required to perform veterinary acts and the characteristics of veterinarians. In the first model (Equation 4), the dependent variable (*Y*) is the weighted time of CA acts, and in the second model (Equation 5), the dependent variable is the weighted time of FPA acts. The explanatory variables represent the variables characterizing the veterinarians: status, gender, and the class of year experience.

(4) $$\begin{array}{l} \text{Weighted time of CA acts}=\;\beta_{1\;}\text{Status}+\beta_{2\;}\text{Gender}\\ \;+\beta_{3\;}\text{Experience}+\;\epsilon \end{array}$$

(5) $$\begin{array}{l} \text{Weighted time of FPA acts}=\beta_{1\;}\text{Status}+\beta_{2\;}\text{Gender}\\ \;+\beta_{3}\text{Experience}+\;\epsilon \end{array}$$

## Results

### Principal Component Analysis

The PCA was performed using two dimensions accounting for a total of 43% of the data variance, with the first and second dimensions explaining 22.5 and 20.7% of the inertia, respectively. None of the remaining dimensions explained more than 15% of the inertia (Table 4).

**Table 4:** Eigenvalues and percentages of variance of the first nine dimensions obtained in the principal component analysis; they represent the amount of variability in veterinary practices explained in each synthetic dimension.

	**Eigenvalue**	**Percentage of variance**	**Cumulative percentage of variance**
Dim 1	2.028	22.541	22.541
Dim 2	1.851	20.715	43.117
Dim 3	1.358	14.946	58.052
Dim 4	0.989	10.995	69.048
Dim 5	0.985	10.946	79.994
Dim 6	0.708	7.872	87.867
Dim 7	0.571	6.347	94.215
Dim 8	0.470	5.223	99.439
Dim 9	0.050	0.560	100.000

**Figure 2:**
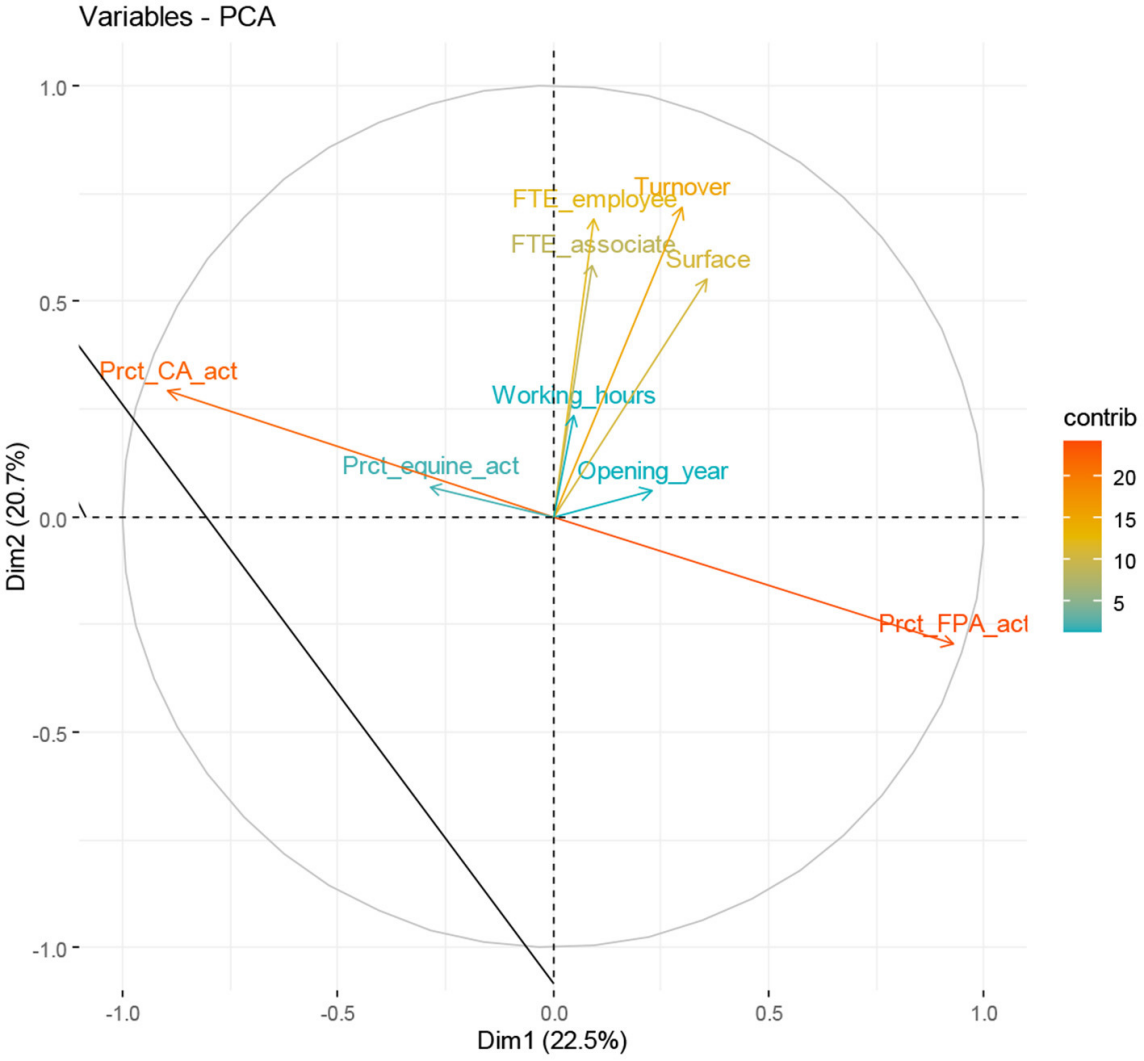
The contribution of the variables characteristic of the veterinary practices on the first two dimensions representing a variance of 43%.

The correlation circle (Figure 2) shows that:

(i) The variables that represent the nature of the veterinary activity: “percentage of CA acts” and “percentage of FPA acts” are strongly correlated to the first dimension (*r* ≥ 0.72).
(ii) The variables that represent the economic performance of veterinary practices: “turnover” and “number of FTE associates and employees” are correlated to the second dimension (*r* ≥ 0.54).

The contribution of variables to the formation of axes follows the correlation of the variables with the axes. The more the color of contribution tends toward red, the higher the contribution of the variable (Figure 2), i.e., the contribution of variables related to the nature of the veterinary activity (CA/FPA acts) is very high compared to other variables (Contrib > 0.8).

For the second dimension, the turnover variable has a high contribution (Contrib = 0.54), then the number of FTE employee (Contrib = 0.50), followed by the surface (Contrib = 0.41) and the number of FTE associate (Contrib = 0.35).

The variables “number of working hours of the veterinary practices, percentage of CA and FPA acts, and the opening year of the veterinary practices” are not well-represented in the first two dimensions (low Contrib). However, they contribute to the formation of the (i) third dimension (the case of the variable year of opening), (ii) the fourth dimension (the case of the variable percentage of equine activity), and (iii) the fifth dimension (the case of the variable of opening hours of the veterinary practices).

### Hierarchical Cluster Analysis

**Figure 3:**
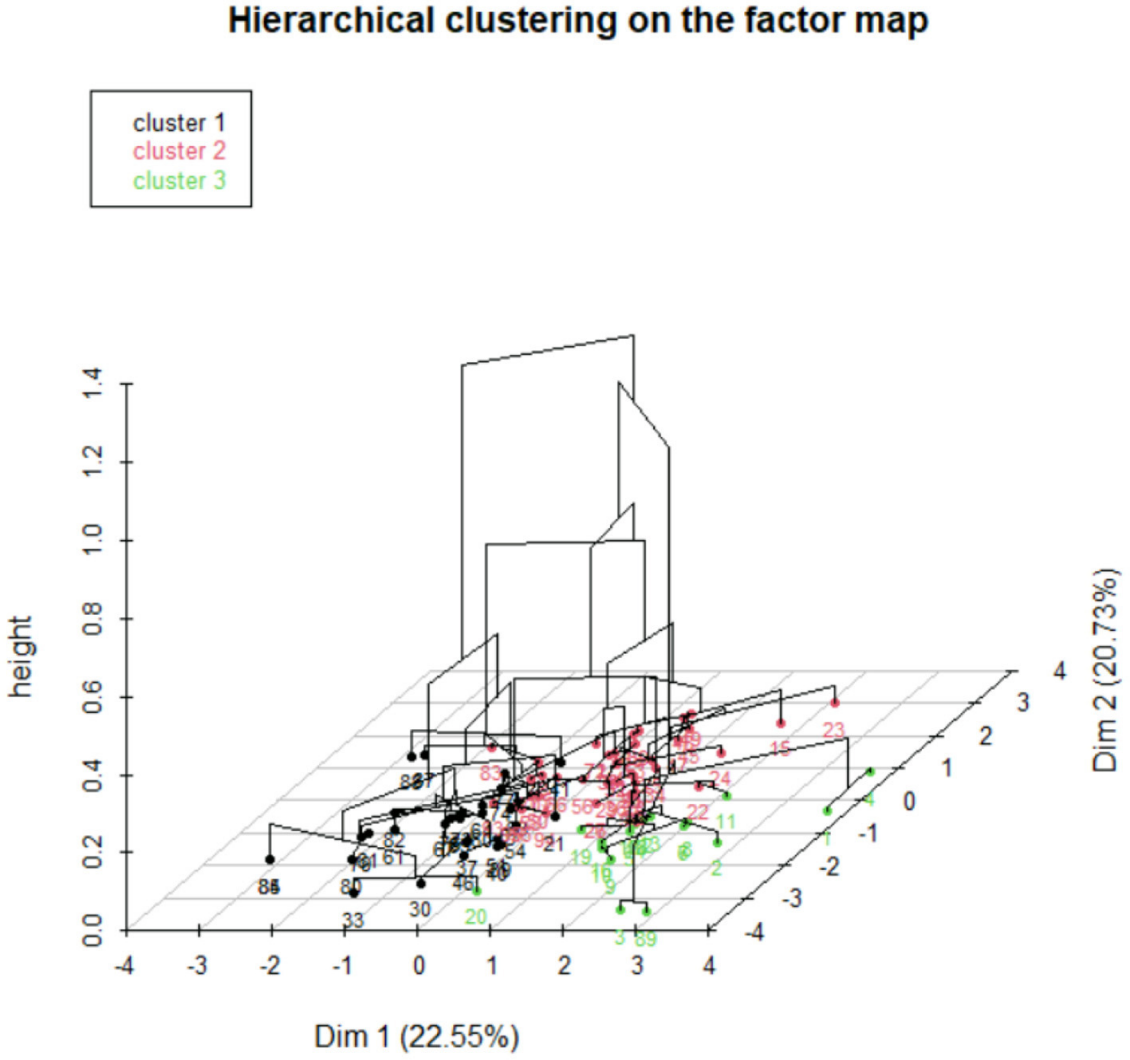
Representation of three clusters of veterinary practices through hierarchical classification.

Based on the decrease in within group inertia, the consolidated HCA revealed three clusters of veterinary practices identified as cluster 1 (*n* = 31), cluster 2 (*n* = 49), and cluster 3 (*n* = 16) (Figure 3).

The variables that are under or overrepresented (*p*-value <0.05) in the different clusters are presented in Table 5.

**Table 5:** Characteristics of the clusters of veterinary practices obtained with the PCA and the consolidated HCA.

	**Cluster 1**		**Cluster 2**		**Cluster 3**	
	**Mean**	**SD**	**Mean**	**SD**	**Mean**	**SD**
Percentage of CA acts (%)	50	10	40	10	20	9
Percentage of FPA acts (%)	30	10	40	10	75	11
Turnover (€)	800,000	300,000	1,000,000	400,000	966,000	393,222
FTE associate (FTE)	2.2	1.3	3.4	1.6	2.2	1.6
FTE employee (FTE)	0.9	0.7	2.9	1.3	1.1	0.6
Structure surface (m^2^)	100	70	200	80	182	120
Structure working hours (h)	51	50	50	3	60	8

### Regression 1: the Association Between the Turnover and the Characteristics of Veterinary Practices

The first model [Table 6, (1)] shows that the turnover (expressed in log) was positively associated with the following: log (working hours), log (surface), FTE associate, FTE employee, and the ratio of CA_FPA acts. An increase of 1% of the surface was associated with a 0.45% increase in turnover. An increase of 1% of the working hours was associated with a 2.73% increase in turnover. An additional FTE associate in the veterinary practice was associated with a 12% increase in turnover. An additional FTE employee in the veterinary practice was associated with an 8% increase in turnover. A one-unit increase of the ratio of CA_FPA acts was associated with a 6% increase in turnover.

**Table 6:** Results of regression models of the association between the variables characteristic of veterinary practices and the turnover.

	**Dependent variable : log (turnover)**		
	**(1)**	**(2)**	**(3)**
Log (surface)	0.4549^***^ (0.1016)	0.4178^***^ (0.1041)	0.4105^***^ (0.1030)
Log (working hours)	2.7370^***^ (0.1383)	2.7475^***^ (0.1507)	2.8359^***^ (0.1274)
FTE associate	0.1251^***^ (0.0343)	0.1229^***^ (0.0348)	0.1241^***^ (0.0351)
FTE employee	0.0851^**^ (0.0313)	0.0639^*^ (0.0310)	0.0864^**^ (0.0320)
Ratio CA_FPA acts	0.0640^**^ (0.0370)		
Percentage of CA acts		0.0184^**^ (0.0044)	
Percentage of FPA acts			0.0013 (0.0027)
*R*^2^	0.88	0.88	0.89
AIC	70.39000	68.61533	71.48973

* *P < 0.1;*

** *P < 0.05;*

*** *P < 0.01*.

The second [Table 6, (2)] and third [Table 6, (3)] sub-models have stable coefficients for all the four variables [log (working hours), log (surface), FTE associate, and FTE employee]. When adding the percentage of CA acts instead of the ratio of CA_FPA acts, a one-unit increase of the percentage of CA acts was associated with a 1.84% increase in turnover [Table 6, (2)]. When adding the percentage of FPA acts instead the ratio of CA_FPA acts, no positive association between the turnover and the percentage of FPA acts was observed.

The dependent variable is the same for all three models, and in order to know which model is the best, we calculated a model selection indicator (Akaike's information criterion; AIC). When comparing models fitted by maximum likelihood to the same data, the smaller the AIC, the better the fit. The second model has the lowest AIC (AIC = 68.61533) and therefore the best fit [Table 6 (2)].

### Regression 2: the Association Between the Weighted Price and the Characteristics of Veterinary Practices

The results of the model [Table 7, (1)], where the dependent variable represents the weighted price of CA acts, show that the weighted price (expressed in log) was positively associated with the following: working hours, FTE associate, ratio of CA_FPA acts, and the median level life. An increase of 1% of the working hours was associated with a 0.71% increase in weighted price of CA acts. An additional associate in the veterinary practice was associated with a 4.6% increase in weighted price of CA acts. A one-unit increase of the ratio of CA–FPA acts was associated with a 6.5% increase in weighted price of CA acts. A one-unit increase of the median level life was associated with a 0.02% increase in weighted price of CA acts.

**Table 7:** Results of the regression models of the association between the variables characteristic of veterinary practices, the median standard living and the weighted price of CA acts (1) and weighted price of FPA acts (2).

	**Dependent variable**	
	**(1) Log (weighted price CA acts)**	**(2) Log (weighted price FPA acts)**
Log (structure surface)	0.002 (0.0002)	0.001 (0.0002)
Log (structure working hours)	0.7162^***^ (0.0704)	0.202^***^ (0.049)
FTE associate	0.0459^*^ (0.0214)	0.0297 (0.0206)
FTE employee	0.0399 (0.0224)	0.0357 (0.0216)
Ratio of CA_FPA acts	0.0652^***^ (0.0256)	0.0012 (0.0998)
Median level life	0.0002^***^ (0.00001)	0.0001^***^ (0.00003)
*R*^2^	0.80	0.80

* *P < 0.1;*

*** *P < 0.01*.

In the model [Table 7, (2)], the weighted price of FPA acts was associated with working hours of the veterinary practice and the median of level life. An increase of 1% of the working hours was associated with 0.2% increase in the weighted price of FPA acts, and a one-unit increase of the median level life was associated with a 0.01% increase in the weighted price of FPA acts.

### Regression 3: the Association Between the Weighted Time Required to Perform Veterinary Acts and the Characteristics of Veterinarians

The results of the models show that the status of veterinarians was positively associated with the weighted time of CA acts [Table 8, (1)] and FPA acts [Table 8, (2)]. The weighted time of execution of CA acts of an associate is on average 32 min. Therefore, the collaborator (23 min) takes less time compared to an associate with a difference of 9 min, and the employee (37 min) takes 5 min more compared to an associate [Table 8, (1)]. The weighted time to perform FPA acts of an associate is on average 34 min. The collaborator and the employee take almost the same time (35 min).

**Table 8:** Results of the models of the association between the variables characteristic of the veterinarians and the weighted time to perform CA acts (1) and FPA acts (2).

	**Dependent variable**	
	**(1) Time CA acts**	**(2) Time FPA acts**
Relevel (status, ref = “associate”) associate	32.485^***^ (5.092)	34.012^***^ (3.532)
Relevel (status, ref = “associate”) collaborator	23.2759^**^ (11.6403)	35.7186^***^ (5.8762)
Relevel (status, ref = “associate”) employee	37.1911^***^ (6.5598)	35.7215^***^ (3.3115)
Relevel (gender, ref = “woman”) men	−1.8778 (5.3534)	0.4250 (2.7025)
Class year experience > 15	−1.1128 (5.9432)	−3.6376 (3.0002)
Class year experience 10–15	−4.5796 (7.9516)	−4.0680 (4.0141)
Class year experience 5–10	−2.9150 (6.7062)	−5.0688 (3.3854)
*R*^2^	0.89	0.88

** *P < 0.05;*

*** *P < 0.01*.

## Discussion

A veterinary practice combines various factors of production in order to produce goods and/or services for their clients. We showed that the turnover of the veterinary practices sampled is mainly associated and increased with (i) the number of working hours of the veterinary practice, (ii) the number of associates and employees working in the veterinary practice, (iii) the surface of the veterinary practice, and (iv) the percentage of CA acts.

We also investigated the pricing of acts among practices in order to understand on which basis the veterinary practices define the price of veterinary acts. The weighted price for CA acts was mainly associated with the number of FTE associate, the number of working hours, the ratio of CA–FPA acts, and the median of the inhabitants' standard of living in the practice catchment area. The more the veterinary practice remains open, the more the prices increase. The fact that the ratio of CA–FPA acts and especially the percentage of CA acts was associated with the weighted price of CA acts means that the more the veterinary practice makes CA acts, the more the prices increase and the richer the inhabitants are and the more they are ready to pay for medical services.

We tested whether the time spent on each activity differed between veterinarians, and we showed that collaborators perform CA acts faster than associates do. This may be explained by the fact that the collaborators are paid on a fee-for-service basis, and therefore, to generate a large margin, it is in their interest to do several acts in 1 day, then they are fast compared to associates and employees. The employees work slower than associates do to perform CA acts; this may be explained by their level of experience. However, the time of execution of FPA acts is almost similar for all veterinarians, with a difference of only 1 min less for associates, compared to collaborators and employees.

This study shows the association between the internal resources (surface, number of employees and associates, and working hours), the nature of veterinary acts (CA acts/FPA acts), and the turnover of veterinary practices. The economic performance of the veterinary practices is relatively associated with the nature of veterinary acts. In a recent publication, we calculated the average profits of veterinary practices, arising from veterinary acts and sales of drugs and materials (2). We have shown that the profit generated by CA acts is higher than the profit generated by FPA acts. However, in the veterinary sector, human resource represents the main indirect expenses, and the procedure used for allocating their related costs could have a large impact on the profit of the activities (2).

Profitability of veterinary practices could be linked with the attractiveness of young graduated veterinarians, as well as leading to a switch of activities in favor of CA in mixed practices. The French National Order of Veterinarians showed that the number of registered veterinarians practicing CA acts increased (+1.5%) with 15,176 new veterinarians, whereas a decrease of 135 veterinarians (−2%) was observed for the same period (10). Though this phenomenon is likely multifactorial, the profitability of activity and remuneration remain important components of decision making and choices of young veterinarians.

Veterinarians may increase or maintain their profit by increasing the revenues of medical acts and a rational allocation of time. Time required for activities is all the more important to evaluate the marginal profitability of each activity given a context this permits new perspectives for resource allocation and long- or short-term projection of firm trajectory (8).

From the hierarchical classification, we have shown three groups of veterinary practices: (i) a group with CA act dominance with a lower turnover than the other groups, (ii) a group with the same share of CA and FPA acts and it is the group that generates a high turnover, and (iii) a third group with FPA act dominance, which works more than the others and has a high turnover. From these three groups, it can be concluded that veterinary practices have different production strategies (more CA than FPA, same, or more FPA than CA).

If we consider veterinary practices as firms run by associates seeking to maximize its profit, the associates will choose whether they want to invest in the veterinary practice (more space, more employees, work more hours…) or keep their usual work pace. This is the case for all firms in market economy that vary considerably in terms of size, profitability, and duration. A firm consists of a manager, which employs capital and labor to produce outputs, and the preferences of managers are different (13). Managers may have different objectives, such as maximizing sales (or total income) subject to profit constraint (13), while Jensen (14) defends the idea that managers may have incentives to grow their firms beyond their optimal size.

The regression models run in the present work used dependent variables in logarithmic form (the case of the dependent variables: turnover and weighted price), since the distribution of these variables is not normal.

We opted for a sequential regression for the first regression model, to highlight the intrinsic impact of the companion animal and food-producing acts, respectively [models (2) and (3)], in order to better analyze the determinants of turnover. The first model [model (1)] showed a positive association between the ratio of CA_FPA acts and the turnover. In order to know if the increase of turnover was due to an increase of CA acts or a decrease of FPA acts, two other sub-models were applied. We included the percentage of CA acts as an explanatory variable instead of the ratio of CA_FPA acts and the percentage of FPA acts instead of the ratio of CA_FPA acts. To determine the best out of the three models run in regression 1 (Table 6), we calculated the indicator of selection of the best model (AIC) and we found that the best model is the second one, with the percentage of CA acting as an explanatory variable instead of ratio of CA–FPA acts with a low AIC. For the three models, the coefficients of the explanatory variables remain stable. Two explanatory variables (surface and number of working hours) were linearized in order to smooth the order of magnitude of these variables. We calculated the relative elasticity between these two variables and found that the variable “working hours” has a more important effect than the surface. This can be explained by the fact that the working time of the veterinary practices is a short-term parameter, while investing in the structure and increasing the surface is a long-term parameter, which represents a high fixed cost with depreciation.

In the regression models 2 and 3, the dependent variable is different in the regression models ran; therefore, we cannot compare two models with two different dependent variables.

One limitation of our study is the small number of observations, which limits the robustness of our econometric estimates. We chose to recruit a convenience sample of veterinarians in this study, to increase the amount of information collected. The surveys lasted an average of 30 min and we captured many that may have an effect on the turnover, the price of veterinary act, and the time required to perform veterinary acts. In our sample, the average turnover is 1,298,099 € and the average number of FTE associate and employee are 2.83 and 2, respectively. The average number of FTE associate and employee in mixed practice in France is 2.44 and 2.33, respectively (10).

Our results have to be extrapolated to the French veterinary population carefully; however, with our selected variables, we quantified how the turnover of veterinary practices varied and what veterinary practices are used as a baseline for setting the prices of FPA and CA acts. Further research conducted on a larger sample and including managerial strategy of the associates would allow confirming the results of our exploratory analysis, where we develop a methodological framework to evaluate the economic performance of veterinary practices.

## Conclusion

We described and analyzed the characteristics of veterinary practices influencing their economic performance and showed a positive association between the status of the veterinarians and the weighted time required to perform veterinary acts. The present results may help decision making for the veterinary firm management, for instance to determine if it is profitable to invest in the building (more consultation rooms), integrate an associate, or orient toward CA animal acts.

## Data Availability Statement

The raw data supporting the conclusions of this article will be made available by the authors, without undue reservation.

## Author Contributions

IA, GL, and DR contributed to the conception and design of the study. IA conducted the analyses. IA, GL, YN, AF, and DR evaluated results and discussed their implications. IA wrote the first draft of the manuscript. All authors contributed to the article and approved the submitted version.

## Conflict of Interest

The authors declare that the research was conducted in the absence of any commercial or financial relationships that could be construed as a potential conflict of interest.

## References

[1] Institut National de la Statistique et des Études Économiques. (2020). Available online at: https://www.insee.fr/ (accessed March 10, 2021).

[2] Minviel JJ Abdouttalib I Sans P Ferchiou A Boluda C Portal J. Business models of the French veterinary offices in rural areas and regulation of veterinary drug delivery. Prev Vet Med. (2019) 173:104804. 10.1016/j.prevetmed.2019.104804 31683187

[3] Behrouzi F Ma'aram A. Identification and ranking of specific balanced scorecard performance measures for hospitals: a case study of private hospitals in the Klang Valley area, Malaysia. Int J Health Plann Manage. (2019) 34:1364–76. 10.1002/hpm.2799 31025447

[4] Van der Wees PJ Nijhuis-van der Sanden MW van Ginneken E Ayanian JZ Schneider EC WG. Governing healthcare through performance measurement in Massachusetts and the Netherlands. Health Policy. (2014) 116:18–26. 10.1016/j.healthpol.2013.09.009 24138729 PMC4744871

[5] Soysa IB Jayamaha NP Grigg NP. Developing a strategic performance scoring system for healthcare nonprofit organisations. Benchmarking. (2018) 25:3654–78. 10.1108/BIJ-02-2017-0026

[6] Dorward A. Agricultural labour productivity, food prices and sustainable development impacts and indicators. Food Policy. (2013) 39:40–50. 10.1016/j.foodpol.2012.12.003

[7] Belage E Croyle SL Jones-Bitton A Dufour S Kelton DF. A qualitative study of Ontario dairy farmer attitudes and perceptions toward implementing recommended milking practices. J Dairy Sci. (2019) 102:9548–57. 10.3168/jds.2018-15677 31326172

[8] Abdouttalib I Lhermie G Cavex J Ferchiou A RD. Time required by veterinarian to perform veterinary acts in routine: a regression analysis. J Bus Econ. (2020) 11.

[9] Google Maps (2021). Available online at: https://www.google.fr/maps (accessed March 08, 2021).

[10] Atlas Démographique de la Profession Vétérinaire. (2020). Available online at: https://www.veterinaire.fr/ (accessed March 3, 2021).

[11] CoreTeam R. R: A Language and Environment for Statistical Computing. (2017). Available online at: https://www.r-project.org/ (accessed January 20, 2021).

[12] Le S Josse J Husson F. FactoMineR: a package for multivariate analysis. J Stat Softw. (2008) 25:1–18. 10.18637/jss.v025.i01

[13] Fisher MR. Business behavior, value and growth. Econ J. (1962) 72:708–11. 10.2307/2228457

[14] Jensen AC. Agency costs of free cash flow, corporate finance, and takeovers. In: The American Economic Review, Vol. 76, No. 2, Papers and Proceedings of the Ninety-Eighth Annual Meeting of the American Economic Association. Boston, MA (2018). p. 323–329.

