# Supplementary material for: The Association Between French Veterinary Practice Characteristics and Their Revenues and Veterinarian's Time Use
Source: Front Vet Sci. 2021 Jun 11;8:675028. doi: 10.3389/fvets.2021.675028 (PMC8231293; doi:10.3389/fvets.2021.675028)
Supplement: Supplementary file 1 [file Data_Sheet_1.PDF]

Survey Questionnaire

Profitability of the veterinary practice: analysis of contribution margin and work time
Part 1.A: Participating Veterinarian Structure Information

The individual information you provide will remain strictly confidential and will be stored on a secure server. A unique identification number will be assigned for statistical processing. It is possible not to answer a question and to continue the questionnaire. No information will be used to identify individuals.

1/ Type of Health Care practice:

Choose among the three proposals.

- Practice ☐
  (A veterinary care practice organized into a complex of premises with at least one reception area and one examination area)
- Clinic ☐
  (A veterinary health care practice organized into a set of premises including at least a reception area, an examination area, a surgery area, a medical imaging area and a hospitalization area)

2/ Name of the municipality of the main professional domicile of practice (DPE)

3/ Postal code of the commune of the main professional domicile of practice (DPE)

4/ Year of opening of the practice

5/ Practice's activity

- % Companion animal acts :
- % Food-producing acts :
- % Equine acts :
- % others :

6/ Number of full-time equivalent (FTE) veterinarians

UMR ENVT-IHAP Epidémiologie, Economie de la Santé et Santé Publique Vétérinaire
Guillaume Lhermie DVM, MSc, PhD guillaume.lhermie@envt.fr
Maela Payen maela.payen_17@envt.fr

7/ Associates

8/ Employees

9/ Veterinary Assistants

10/ Number of secretarial staff

11/ Number of professional homes of practice

12/ Surface of the structure (in m²)

13/ Number of rooms in the practice

The following rooms are counted as rooms: reception, waiting room if separate from the reception, consultation room, laboratory, surgery room, pharmacy, storage room for cadavers, kennel, cattery, large animal hospitalization, radio room and/or autopsy room, rest room, storage room, administrative room

14/ Number of consulting rooms

15/ Number of surgical rooms

16/ Number of days the practice is open

Example: If the clinic is open Monday to Friday all day, and Saturday morning, the answer is 5.5

17/ Opening hours of the practice

Monday to Friday

Morning Afternoon

Saturday

Morning Afternoon

18/ Percentage of consultation room use per day (estimate)

19/ Legal form of the practice

Choose an answer among these propositions.

Individual exercise ☐

SCM ☐

SCP ☐

SEL (SELARL, SELAFA, SELCA) ☐

SP ☐

20/ Annual turnover € :

21/ Number of livestock units undergoing health monitoring (for main ECD)
Round to thousands.

22/ Number of farms in sanitary follow-up (for main ECD)

23/ Radius of customers (in km)

UMR ENVT-IHAP Epidémiologie, Economie de la Santé et Santé Publique Vétérinaire
Guillaume Lhermie DVM, MSc, PhD guillaume.lhermie@envt.fr
Maela Payen maela.payen_17@envt.fr

Part 1.B: Procedure Prices Information

If the practice does not operate in one of the areas listed below, please do not answer for that area.

Companion animal:

In the following questions, please indicate each time the rates in euros including VAT for the various procedures specified. For surgical procedures and anesthesia, please indicate the rate for a dog of about 10 kg.

1/ Consultation **€ :**

2/ Injection **€ :**

3/ Abdominal ultrasound €

4/ Euthanasia, including anesthesia € :

5/ Vaccine

- CN CHLP **€ :**
- CN CHLPR **€ :**
- CT CRP **€ :**
- CT CRPL **€ :**

6/ Placement of catheter and single infusion € :

7/ Passport € :

8/ Radiography
- 2 images (face and profil) €:

- Additionnal image **€ :**

9/ Electronic identification €: 10/

Blood test (act only) €:

*** 11/ to 18/: anaesthesia included**

11/ Cat castration *€:

12/ Dog castration ***€ :**

13/ Cat ovariectomy* **€ :**

14/ Dog ovariectomy ***€ :**

UMR ENVT-IHAP Epidémiologie, Economie de la Santé et Santé Publique Vétérinaire
Guillaume Lhermie DVM, MSc, PhD guillaume.lhermie@envt.fr
Maela Payen maela.payen_17@envt.fr

15/ Mammectomy * **€ :**

16/ Hysterectomy * **€ :**

17/ EDS Surgery * **€ :**

18/ Descaling * **€ :**

19/ Anesthesia (average price for dog of 10 kg) **€ :**

20/ Blood smear **€ :**

*** 21/ a 23/ : price including blood sampling**

21/ Simple biochemistry (price for 1 parameter)* **€ :**

22/ Complex biochemistry (price per parameter for more than 5 parameters)* **€ :**

23/ Blood counts * **€:**

24/ Test FeLV/FIV* **€ :**

25/ Urine analysis **€ :**

26/ Hospitalization (first day) **€/j :**

27/ Custody supplement **€ :**

<u>Food-producing animals'</u>

In the following questions, please indicate the prices in euros excluding VAT for the various procedures specified.

1/ Consultation **€ :**

2/ Simple calving **€ :**

3/ Complex calving **€ :**

4/ Uterine prolapse **€ :**

5/ Diagnosis of gestation (ultrasound) **€ :**

6/ No delivery **€ :**

7/ Trimming (one foot or first foot)**€ :**

8/ Visit/ Blood test purchase (first animal) **€ :**

UMR ENVT-IHAP Epidémiologie, Economie de la Santé et Santé Publique Vétérinaire
Guillaume Lhermie DVM, MSc, PhD guillaume.lhermie@envt.fr
Maela Payen maela.payen_17@envt.fr

9/ Euthanasia **€ :**

10/ Blood test **€ :**

11/ Autopsy of a calf **€ :** 12/

Herd monitoring **€/h** :13/

Expertise **€/h :**

14/ Infusion **€ :**

15/ Caesarean section cow **€ :**

16/ Abomasal operation **€ :**

17/ Caesarean of sheep **€ :**

18/ Coproscopy **€ :**

19/ Bacteriology of milk **€ :**

20/ Hospitalization of a calf **€/j :**

21/ Displacement **€/km :**

22/ Custody supplement **€ :**

Equine :

In the following questions, please indicate the prices in euros including VAT for the various procedures specified.

1/ Consultation **€ :**

2/ Naso-gastric tube **€ :**

3/ Ultrasound **€ :**

4/ Rolling **€ :**

5/ Perfusion **€ :**

UMR ENVT-IHAP Epidémiologie, Economie de la Santé et Santé Publique Vétérinaire
Guillaume Lhermie DVM, MSc, PhD guillaume.lhermie@envt.fr
Maela Payen maela.payen_17@envt.fr

UMR ENVT-IHAP Epidémiologie, Economie de la Santé et Santé Publique Vétérinaire
Guillaume Lhermie DVM, MSc, PhD guillaume.lhermie@envt.fr
Maela Payen maela.payen_17@envt.fr
