# Supplementary material for: The Association Between French Veterinary Practice Characteristics and Their Revenues and Veterinarian's Time Use
Source: Front Vet Sci. 2021 Jun 11;8:675028. doi: 10.3389/fvets.2021.675028 (PMC8231293; doi:10.3389/fvets.2021.675028)
Supplement: Supplementary file 2 [file Data_Sheet_2.PDF]

Survey Questionnaire

Profitability of the veterinary activity: analysis of the contribution margin and working hours

Part 2.A : Participating veterinarian information

The individual information you provide will remain strictly confidential and will be stored on a secure server. A unique identification number will be assigned for statistical processing. It is possible not to answer a question and to continue the questionnaire. It is possible to go back and make changes

1/ You are:

- ➔ A man ☐
- ➔ A woman ☐

2/ Age

3/ Year of graduation

4/ Current department of practice

5/ Municipality of practice
6/ Postal code of the practice

7/ You are actually

- ➔ In individual exercice ☐
- ➔ Associate ☐
- ➔ Employee ☐
- ➔ Collaborator ☐

8/ Net annual income before income tax

- ➔ <25,000€ ☐
- ➔ Between 25,000€ and 35,000€ ☐
- ➔ Between 25,000€ and 35,000€ ☐
- ➔ Between 35,000€ and 45,000€ ☐
- ➔ Between 45,000€ and 55,000€ ☐
- ➔ Between 55,000€ and 65,000€ ☐
- ➔ Between 65,000€ and 75,000€ ☐
- ➔ Between 75,000€ and 85,000€ ☐
- ➔ Between 85,000€ and 95,000€ ☐
- ➔ >95,000€ ☐

UMR ENVT-IHAP Epidémiologie, Economie de la Santé et Santé Publique Vétérinaire
Guillaume Lhermie DVM, MSc, PhD guillaume.lhermie@envt.fr
Maela Payen maela.payen_17@envt.fr

Part 2.B: Participating Veterinarian's Own Time

In the rest of the questionnaire, ''working-time '' means the actual time spent in the practice (including all management and other activities associates with the operation of the practice), excluding on-call duty. In case of daily or weekly variations, please provide an average value.

1/ Number of half-days worked per week

2/ Number of hours worked per week

3/ Number of night shifts per week

4/ Number of day shifts per week

5/ Number of overtime hours worked per week

6/ Number of weeks off per year

7/ Number of holiday and weekend shifts per year

8/ Number of hours in a veterinary professional activity or related to the veterinary professional activity outside the practice, in 2019

Corresponds to an inspection in a slaughterhouse, teaching, expertise, ordinal or union representation

9/ In 2019, how many hours per week, not included in your work time count, did you spend on average on clinic management activities?

10/ Average number of patients seen per half day

UMR ENVT-IHAP Epidémiologie, Economie de la Santé et Santé Publique Vétérinaire
Guillaume Lhermie DVM, MSc, PhD guillaume.lhermie@envt.fr
Maela Payen maela.payen_17@envt.fr

Part 2.C: Time spent on different activities

<u>In the canine consultation room:</u>

1/ Time devoted to a follow-up canine consultation consisting of a basic clinical examination

Mean (min) :	Min (min):	Max (min) :

For the rest of the questions, the times correspond to the time spent outside the basic consultation.

2/ Time spent on a blood test

Mean (min) :	Min (min):	Max (min) :

3/ Time of a euthanasia, including anesthesia

Mean (min) :	Min (min):	Max (min) :

4/ Time of infusion placement

Mean (min) :	Min (min):	Max (min) :

5/ Time of an abdominal ultrasound since the preparation of the animal

Mean (min) :	Min (min):	Max (min) :

6/ Time of abdominal X-ray 2 images (front and side), including preparation time

Mean (min) :	Min (min):	Max (min) :

<u>In the operating room in canine:</u>

1/ Time of a cat castration

Mean (min) :	Min (min):	Max (min) :

2/ Time of a dog castration

Mean (min) :	Min (min):	Max (min) :

3/ Time of an ovariectomy cat

Mean (min) :	Min (min):	Max (min) :

UMR ENVT-IHAP Epidémiologie, Economie de la Santé et Santé Publique Vétérinaire
Guillaume Lhermie DVM, MSc, PhD guillaume.lhermie@envt.fr
Maela Payen maela.payen_17@envt.fr

4/ Time of an ovariectomy for dogs

Mean (min) :	Min (min):	Max (min) :

5/ Time of a mammectomy for dogs

Mean (min) :	Min (min):	Max (min) :

6/ Time of a dog hysterectomy

Mean (min) :	Min (min):	Max (min) :

7/ Time of a descaling

Mean (min) :	Min (min):	Max (min) :

8/ Time of a dog's anesthesia

From restraint to effectiveness.

Mean (min) :	Min (min):	Max (min) :

9/ Time of SDTE surgery in a dog

Mean (min) :	Min (min):	Max (min) :

<u>In the laboratory or hospital:</u>

1/ Time of a blood smear analysis

Mean (min) :	Min (min):	Max (min) :

2/ Time of a simple biochemistry 10 parameters

From realization to analysis.

Mean (min) :	Min (min):	Max (min) :

3/ Time of a FeLV and FIV test

Mean (min) :	Min (min):	Max (min) :

UMR ENVT-IHAP Epidémiologie, Economie de la Santé et Santé Publique Vétérinaire
Guillaume Lhermie DVM, MSc, PhD guillaume.lhermie@envt.fr
Maela Payen maela.payen_17@envt.fr

4/ Time of a urine analysis

Mean (min) :	Min (min):	Max (min) :

5/ Time of a formula count

Mean (min) :	Min (min):	Max (min) :

6/ Time spent with a hospitalized dog during a 24-hour hospitalization

Mean (min) :	Min (min):	Max (min) :

<u>In Medicine and Surgery Ruminants:</u>

1/ Time of a consultation

Mean (min) :	Min (min):	Max (min) :

2/ Time of a single calving

Mean (min) :	Min (min):	Max (min) :

3/ Time of a complex calving

Mean (min) :	Min (min):	Max (min) :

4/ Time of resolution of uterine prolapse in cows

Mean (min) :	Min (min):	Max (min) :

5/ Time of a pregnancy diagnosis in a cow

Mean (min) :	Min (min):	Max (min) :

6/ Time of a caesarean section in a cow

Mean (min) :	Min (min):	Max (min) :

7/ Average time of a lambing

Mean (min) :	Min (min):	Max (min) :

8/ Time of a prophylaxis purchase

UMR ENVT-IHAP Epidémiologie, Economie de la Santé et Santé Publique Vétérinaire
Guillaume Lhermie DVM, MSc, PhD guillaume.lhermie@envt.fr
Maela Payen maela.payen_17@envt.fr

Mean (min) :	Min (min):	Max (min) :

9/ Time of a cow euthanasia

Mean (min) :	Min (min):	Max (min) :

10/ Time of a calf autopsy

Mean (min) :	Min (min):	Max (min) :

11/ Time of a bovine herd reproduction follow-up (min/ animal)

Mean (min) :	Min (min):	Max (min) :

12/ Time of a left abomasal displacement operation in cows

Mean (min) :	Min (min):	Max (min) :

13/ Average time of an infusion in a cow

Mean (min) :	Min (min):	Max (min) :

14/ Time of a caesarean section ewe

Mean (min) :	Min (min):	Max (min) :

15/ Time of a milk bacteriology in the office

Mean (min) :	Min (min):	Max (min) :

16/ Time of a coproscopy to be analyzed

Mean (min) :	Min (min):	Max (min) :

17/ Time spent on a calf during a 24-hour hospitalization

Mean (min) :	Min (min):	Max (min) :

<u>In Equine Medicine:</u>

1/ Time of a consultation

UMR ENVT-IHAP Epidémiologie, Economie de la Santé et Santé Publique Vétérinaire
Guillaume Lhermie DVM, MSc, PhD guillaume.lhermie@envt.fr
Maela Payen maela.payen_17@envt.fr

Mean (min) :	Min (min):	Max (min) :

2/ Time of a nasogastric tube in horses

Mean (min) :	Min (min):	Max (min) :

3/ Time of an ultrasound in a mare

Mean (min) :	Min (min):	Max (min) :

4/ Time of a foaling

Mean (min) :	Min (min):	Max (min) :

5/ Time of an infusion for a horse

Mean (min) :	Min (min):	Max (min) :

Do you have anything else to add?

Thank you for your participation.

UMR ENVT-IHAP Epidémiologie, Economie de la Santé et Santé Publique Vétérinaire
Guillaume Lhermie DVM, MSc, PhD guillaume.lhermie@envt.fr
Maela Payen maela.payen_17@envt.fr
